# 1108. Severity of Illness among Adults Hospitalized with Respiratory Syncytial Virus Compared with COVID-19 and Influenza — IVY Network, 25 Hospitals, 20 U.S. States, January 31, 2022 – April 11, 2023

**DOI:** 10.1093/ofid/ofad500.081

**Published:** 2023-11-27

**Authors:** Katharine Yuengling, Diya Surie, Jennifer DeCuir, Yuwei Zhu, Manjusha Gaglani, Adit A Ginde, Kevin Gibbs, Matthew Prekker, Amira Mohamed, Nicholas Johnson, Ithan Peltan, William Bender, Christopher Mallow, Jennie H Kwon, Adam S Lauring, Cristie Columbus, Ivana Vaughn, Basmah Safdar, James Chappell, Adrienne Baughman, Sydney A Swan, Cassandra Johnson, Meredith L McMorrow, Wesley Self, Emily T Martin

**Affiliations:** GDIT/Centers for Disease Control and Prevention, Los Angeles, CA; Centers for Disease Control and Prevention, Atlanta, Georgia; Centers for Disease Control and Prevention, Atlanta, Georgia; Vanderbilt University, Nashville, Tennessee; Baylor Scott & White Health, Temple, TX; University of Colorado, Aurora, Colorado; Wake Forest University Baptist Medical Center, Winston-Salem, North Carolina; Hennepin County Medical Center, Minneapolis, Minnesota; Montefiore medical center, Bronx, New York; University of Washington School of Medicine, Seattle, Washington; Intermountain Medical Center and University of Utah, Salt Lake City, Utah; Emory University School of Medicine, Atlanta, Georgia; University of Miami, Miami, Florida; Washington University - School of Medicine, St. Louis, MO; University of Michigan, Ann Arbor, MI; Baylor Scott & White Health - Dallas, Dallas, Texas; Henry Ford Health, Detroit, Michigan; Yale University School of Medicine, New Haven, Connecticut; Vanderbilt University Medical Center, Nashville, Tennessee; Vanderbilt University Medical Center, Nashville, Tennessee; Vanderbilt University Medical Center, Nashville, Tennessee; Vanderbilt University Medical Center, Nashville, Tennessee; CDC/NCIRD/CORVD/SPB, Atlanta, GA; Vanderbilt University Medical Center, Nashville, Tennessee; University of Michigan, Ann Arbor, MI

## Abstract

**Background:**

Data on the severity of RSV-associated hospitalizations in adults compared with COVID-19- and influenza-associated hospitalizations are limited. We compared characteristics and clinical outcomes among adults hospitalized with RSV vs. COVID-19, and RSV vs. influenza.

**Methods:**

Adults aged ≥18 years hospitalized with acute respiratory illness (ARI) who tested positive for RSV, SARS-CoV-2, or influenza by nucleic acid amplification or antigen test were enrolled at 25 hospitals in 20 U.S. states participating in the Investigating Respiratory Viruses in the Acutely Ill (IVY) Network during January 31, 2022 to April 11, 2023. Patients with respiratory viral co-infections were excluded. Clinical data were abstracted from medical charts. Advanced respiratory support was defined as receipt of high flow nasal cannula, non-invasive ventilation, or invasive mechanical ventilation (IMV) during hospitalization. Characteristics were compared using Chi-square tests. Severe illness was compared for RSV vs. COVID-19 and RSV vs. influenza using multivariable logistic regression models adjusted for age, sex, race and ethnicity, number of chronic medical condition categories, admission date, and geographic region.

**Results:**

Of 8,334 hospitalized adults included, 6% tested positive for RSV (n=478), 80% for SARS-CoV-2 (n=6,664), and 14% for influenza (n=1,192). Median age of patients with RSV was 65 years (IQR = 53–75), similar to patients with COVID-19 and influenza (**Table 1**). Shortness of breath was more frequently reported by patients with RSV compared to patients with COVID-19 (78% vs. 62%, *P*< 0.0001) and influenza (78% vs 72%, *P*=0.005). Patients with RSV were more likely to be hypoxemic compared to those with COVID-19 (aOR 1.92, 95% CI = 1.52–2.42) (**Table 2**). Patients with RSV were also more likely to receive advanced respiratory support compared to those with COVID-19 (aOR 1.88, 95% CI = 1.51–2.33) or influenza (aOR 1.83, 95% CI = 1.4–2.4); however, use of IMV did not differ between these groups.

**Figure:**
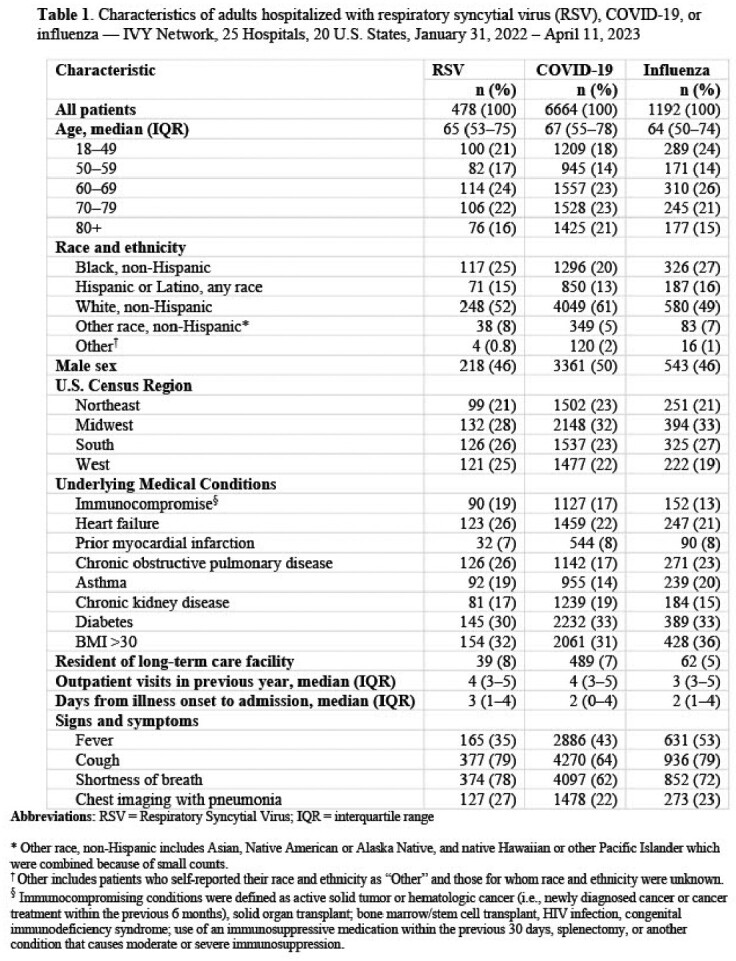

**Figure:**
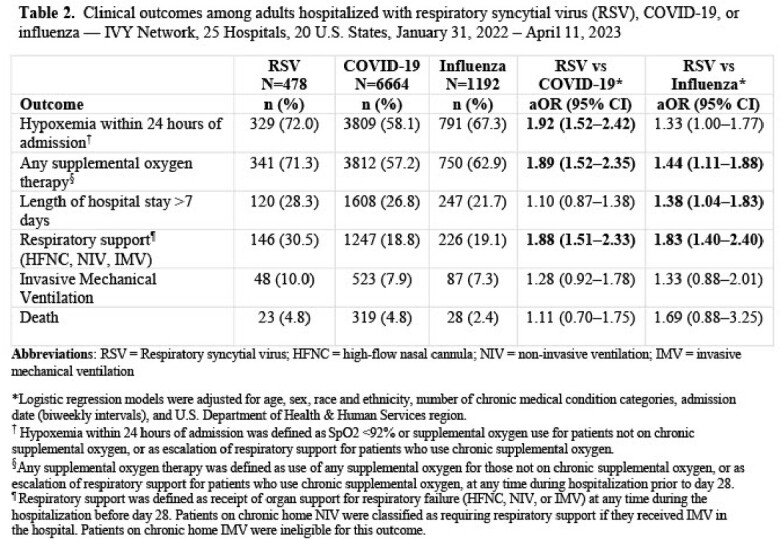

**Conclusion:**

In this prospective, multicenter network, prevalence of RSV among adults enrolled with ARI was lower than patients with COVID-19 and influenza; however, adults hospitalized with RSV experienced more severe respiratory illness compared to patients hospitalized with COVID-19 or influenza.

**Disclosures:**

**Adit A. Ginde, MD, MPH**, AbbVie: Grant/Research Support|Faron Pharmaceuticals: Grant/Research Support **Ithan Peltan, MD**, Asahi Kasei Pharma: Institutional support|Regeneron: Institutional support **Adam S. Lauring, MD, PhD**, Roche: Advisor/Consultant|Sanofi: Advisor/Consultant **Emily T. Martin, PhD, MPH**, Merck: Grant/Research Support

